# SPARCoC: A New Framework for Molecular Pattern Discovery and Cancer Gene Identification

**DOI:** 10.1371/journal.pone.0117135

**Published:** 2015-03-13

**Authors:** Shiqian Ma, Daniel Johnson, Cody Ashby, Donghai Xiong, Carole L. Cramer, Jason H. Moore, Shuzhong Zhang, Xiuzhen Huang

**Affiliations:** 1 Department of Systems Engineering and Engineering Management, The Chinese University of Hong Kong, Shatin, N.T. Hong Kong; 2 Molecular Biosciences Program, Arkansas State University, Jonesboro, Arkansas 72467, United States of America; 3 Department of Pharmacology and Toxicology and the Cancer Center, Medical College of Wisconsin, Milwaukee, Wisconsin 53226, United States of America; 4 Arkansas Bioscience Institute and Department of Biological Sciences, Arkansas State University, Jonesboro, Arkansas 72467, United States of America; 5 The Geisel School of Medicine, Dartmouth College, Lebanon, New Hampshire 03756, United States of America; 6 Department of Industrial and Systems Engineering, University of Minnesota, Minneapolis, Minnesota 55455, United States of America; 7 Department of Computer Science, Arkansas State University, Jonesboro, Arkansas 72467, United States of America; Harbin Medical University, CHINA

## Abstract

It is challenging to cluster cancer patients of a certain histopathological type into molecular subtypes of clinical importance and identify gene signatures directly relevant to the subtypes. Current clustering approaches have inherent limitations, which prevent them from gauging the subtle heterogeneity of the molecular subtypes. In this paper we present a new framework: SPARCoC (Sparse-CoClust), which is based on a novel Common-background and Sparse-foreground Decomposition (CSD) model and the Maximum Block Improvement (MBI) co-clustering technique. SPARCoC has clear advantages compared with widely-used alternative approaches: hierarchical clustering (Hclust) and nonnegative matrix factorization (NMF). We apply SPARCoC to the study of lung adenocarcinoma (ADCA), an extremely heterogeneous histological type, and a significant challenge for molecular subtyping. For testing and verification, we use high quality gene expression profiling data of lung ADCA patients, and identify prognostic gene signatures which could cluster patients into subgroups that are significantly different in their overall survival (with p-values < 0.05). Our results are only based on gene expression profiling data analysis, without incorporating any other feature selection or clinical information; we are able to replicate our findings with completely independent datasets. SPARCoC is broadly applicable to large-scale genomic data to empower pattern discovery and cancer gene identification.

## Introduction

There is significant interest in developing effective computational approaches to study massive genomic profiling data, such as whole-genome gene expression data, of cancer patients. Due to cancer tumor heterogeneity (see [1–5]), which is well-known to the field, it is challenging to analyze the genomic data in order to cluster cancer patients of a certain histological or pathological cancer type into different molecular subgroups (subtypes) of genetic, biological and clinical importance, and identify cancer genes or gene patterns that are directly relevant to distinguish the different subtypes. Research efforts in molecular subtyping and cancer gene signature discovery could empower important medical applications and clinical translations such as molecular diagnosis, prognosis, and personalized medicine.

Recently there are studies in comprehensive molecular characterizations of different cancers, including the breast cancer molecular study [6–9], colorectal cancer (CRC) classification [10], lung cancer adenocarcinoma (ADCA) or squamous cell (SQ) subtyping [11–15]. The molecular subtyping of each of these studies involves the application of a specific clustering or biclustering/co-clustering method. Hierarchical clustering (Hclust) [16], nonnegative matrix factorization (NMF) [17], integrative clustering (iCluster) [18] and ConcensusClusterPlus [19] are the several popular methods currently used in molecular subtyping of these studies of breast cancer, colorectal cancer, or lung cancer etc [6–15].

However, the existing clustering methods [e.g., 16–19] have inherent limitations. They usually work well for distinguishing different histological or pathological types of cancers, but not for distinguishing fine detailed molecular subtypes of a histological heterogeneous cancer type. Also due to the computational challenge in analyzing large genomic data, most current methods choose to use an approximative computational model as the basis. Current approaches usually preprocess the whole-genome data for gene or feature selection; or they rely heavily on clinical information to guide the clustering of cancer patients [11–15]. However, preprocessing of the data may lose the information of important genes or gene patterns associated with the cancer, and being too dependent on clinical information will potentially introduce bias to cancer heterogeneous molecular subtyping. The limitations of current clustering methods will be further discussed in great detail in the next **Methods** Section.

**Fig 1:**
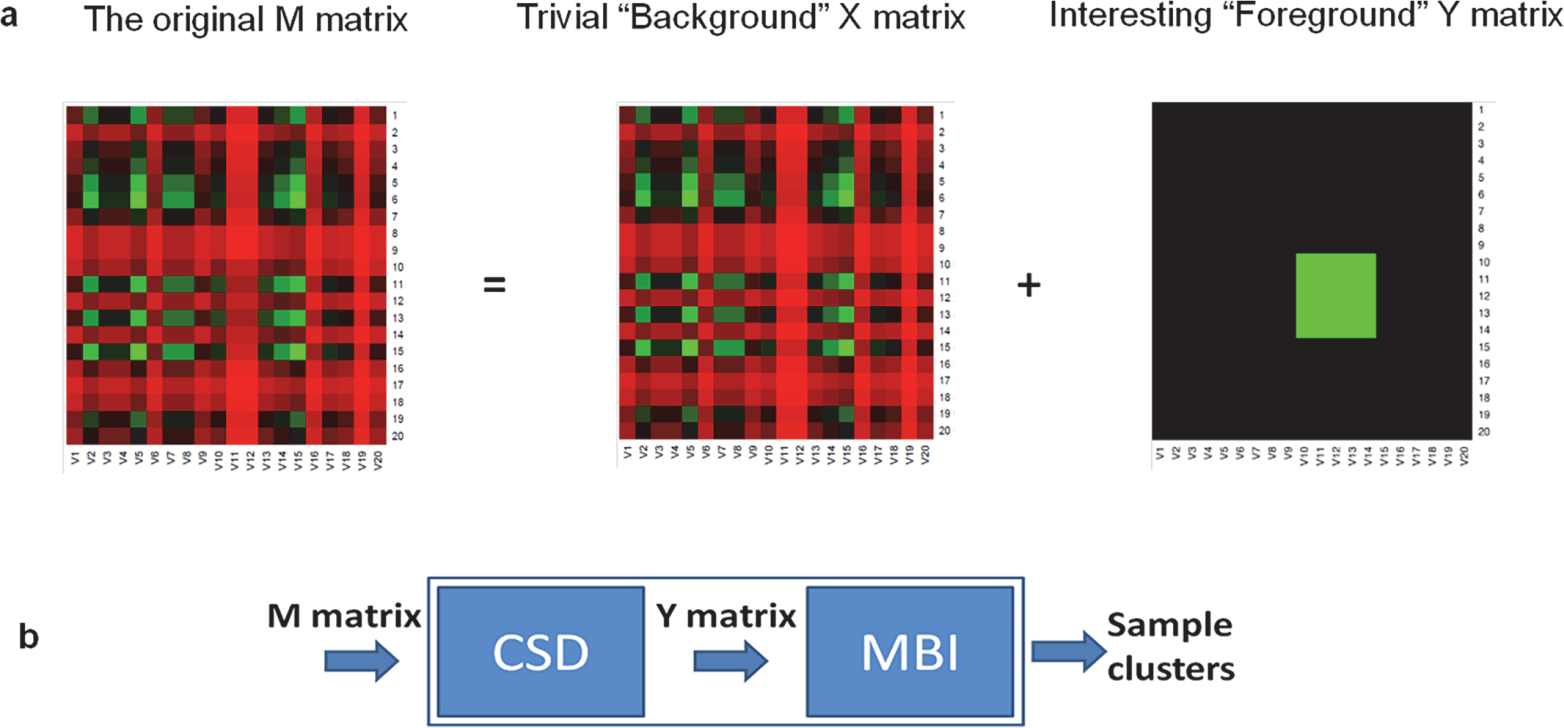
Overview of the new clustering framework. **(a)** An artificial example: Given the input gene expression M matrix, where are the “interesting genes” hidden? (i.e., which are the genes significant for distinguishing the potential different molecular subtypes?) The “interesting” genes are not easily detected from the given M matrix using the current popular clustering methods, e.g., NMF or Hclust. However, we could clearly see the “foreground” (a co-cluster of size 5×5, shown in green of the Y matrix) after the distractive “background” X matrix is removed through the decomposition. The “interesting” genes (rows 10–14) are differentially expressed for samples/columns 10–14 of the Y matrix. **(b)** The new clustering framework. This new framework includes two modules: the common-background and sparse-foreground decomposition (CSD) and the Maximum Block Improvement (MBI) co-clustering. Given an M matrix, the CSD module will decompose M and generate a “foreground” Y matrix; Then, the MBI co-clustering module will work on the Y matrix and output the co-clusters, providing the information of groups of samples and groups of genes that are associated with certain groups of samples. *Our clustering framework conducts clustering by “sparse-foreground” commonality*, *while many current clustering methods usually conduct clustering by “background” commonality*.

Realizing one of the inherent limitations of existing methods is that the common features in the background of the large scale genomic data of cancer patients may obscure the detection of rare but crucial data variations, i.e., the important genomic features defining the fine detailed molecular subtypes of patients. As in imaging processing, when presented with thousands of surveillance pictures of the same background area, if we could remove the distraction of the common background and just focus on the sparse interesting foreground information, we could easily and clearly detect the important patterns. Here, we present SPARCoC (Sparse-CoClust), a new unsupervised clustering framework for discovering molecular patterns and cancer molecular subtypes. The framework is based on a scheme known as common-background sparse-foreground decomposition (CSD) and a technique known as Maximum Block Improvement (MBI) checkerboard co-clustering. This new framework appears to have significant advantages in cancer molecular subtyping and gene signature identification. As we will see later by an example (**Fig. 1A**) that clustering by commonality (which is the philosophy behind almost all existing clustering methods) is fundamentally flawed in the context of cancer molecular subtyping. Instead, the ability to detect the abnormality hidden behind the common background is the core feature of our new approach.

We evaluate this new framework for studying lung adenocarcinoma (ADCA), which is an extreme heterogeneous lung cancer histological type (http://www.cancer.gov/cancertopics/) and which is now a paradigm for molecular subtyping. The studies of lung cancer by many investigators have already shown the feasibility of cancer classification (class discovery and class prediction) based on gene expression profiling of cancer patients [20–24, 13, 14]. Many studies conduct gene expression clustering and search for gene expression signatures; however, the published prognostic gene signatures from different studies have no (or, very few) genes in common [25]. This lack of overlaps may indicate that many genes are involved in lung cancer pathology; equally probably it may also be a consequence of unforeseen pitfalls with clustering based on a small number of genes after trimming and preprocessing.

We apply SPARCoC to analyze whole-genome gene expression profiling data of lung ADCA patients. These datasets (collectively with profiles of more than 600 lung ADCA patient samples) are of high quality and collected with extensive clinical information of the patients. SPARCoC could cluster lung ADCA and stage I lung ADCA patients based on their gene expression profiles into subgroups with significantly different clinical survival outcomes, and the identified gene signatures, when verified using completely independent patient profiling datasets, could separate patients into subgroups of distinct survival outcomes. Specifically, Kaplan-Meier analysis of the overall survival of lung ADCA and stage I lung ADCA patients with the identified 128-gene signature demonstrated that the high- and low-risk groups are significantly different in their overall survival (with p-values < 0.05). Note that the process of lung ADCA patients clustering, gene signature identification, survival analysis and cross-validation is classical to the field (The interested readers are referred to, e.g., [11–15]).

We believe our new framework SPARCoC, when applied to genomic profiling of cancer patients, could potentially lead to new discoveries in the study of cancer molecular subtyping to guide medical treatments and new identification of cancer genes or gene patterns for cancer prognosis or as medical targets.

## Methods

### SPARCoC: a new framework for molecular pattern discovery and cancer gene identification

Our new clustering framework (**Fig. 1**) includes two modules: the common-background and sparse-foreground decomposition (CSD) and the Maximum Block Improvement (MBI) co-clustering. The following is an overview and some brief discussions of the two modules. In the CSD module, the computational model is based on sparse optimization; in the co-clustering module, a block optimization model is adopted. As is discussed in detail in the following, our framework SPARCoC has novel features which make it very effective in molecular pattern discovery, and our computational model is different from the model of robust principal component analysis (RPCA) and other current clustering and biclustering/co-clustering methods.

### An example to illustrate the idea of our clustering framework with CSD decomposition and MBI co-clustering (see Fig. 1)

This example contains three files (see **S1 File** for the details of the example files): M.csv, Y.csv, and X.csv. The background X matrix (size: 20 × 20; entry values ranging from 1~100) is a rank-one matrix randomly generated in MATLAB; the foreground Y matrix (size: 20 × 20 with entry values all set to be 0, except for a co-cluster of size 5×5 with entry values all set to be 10) is added to the background X matrix, we get the M matrix (size: 20 × 20), which is now a rank-two matrix. When given the M.csv (the M matrix), our CSD decomposition model returns exactly X.csv (the X matrix) and Y.csv (the Y matrix) as given (Note that the CSD model we used is the (M3) model, which will be specified later, with K = 1 and noise level δ = 0). When we test the performance of MBI on the Y.csv (the Y matrix), we get the exactly correct co-cluster of size: 5×5. This artificial example shows that our new clustering framework based on the CSD decomposition and the MBI co-clustering can effectively separate the “interesting” foreground information (of interesting genes and interesting samples) from the background information. We would like to point out that even with this simple example, it is hard for other clustering approaches, such as NMF, to correctly separate the interesting samples from the other samples when the M matrix is given.

### The Common-background and Sparse-foreground Decomposition (CSD) module

We used the following two models for common-background and sparse-foreground decomposition: (M1) and (M2).

(Model 1) The model is to write a given matrix M as the sum of three matrices: X, Y and Z, in such a way that M = X + Y + Z, while X is a rank-one matrix in the form of X = x*ι where x is a decision vector and ι is the all-one row vector, and Z is the noise matrix. Specifically, the model in question is

$$\begin{array}{c} \min\left\|Y\right\|\\ s.t.\;x^{*}\;L+Y+Z=M\\ {\left\|Z\right\|}_{F}\leq \delta . \end{array}$$ (M1)

Note that X thus has a common-vector structure in the sense that all the column vectors of X are the same.

It should be pointed out that our common-vector model is theoretically different from the RPCA model proposed in Candes et al. [26] and Chandrasekaran et al. [27]. The main difference is RPCA requires X to be low-rank, but our model (M1) requires X to be a special rank-one matrix. The L_1_ norm in the objective of (M1) naturally promotes the sparsity in matrix Y. Recently, a similar model for imaging background extraction was also considered independently by Li, Ng and Yuan [28] in the context of image processing for applications in video surveillance systems. We solve (M1) by the so-called Alternating Direction Method of Multipliers (ADMM), which is a first-order optimization routine, allowing us to solve very large size models.

(Model 2) Consider gene expression matrices M_k_ of the same dimension m × n, and k = 1, 2, …, K. Index k denotes a given condition. For a given k, matrix M_k_ = (a^k^_ij_)m×n contains the expression level of gene i under time point j, where i = 1, 2, …, m and j = 1, 2, …, n. We can model the background fluctuation of the expression level by a low-rank matrix, and the remaining sparse matrices then reflect the foreground which “shows” the expression of the “interesting” or “active” genes. This information can be used to analyze the relation or correlation among the gene expression level/pattern and type/subtypes. The optimization model of interest is:

$$\begin{array}{c} \min\;\mathrm{rank}\;X+{\Sigma_{i=1}}^{K}\rho_{i}{\left\|Y_{i}\right\|}_{0}\\ s.t.\;X+Y_{i}+Z_{i}=M_{i},\;i=1,\ldots ,K\\ {\left\|Z\right\|}_{F}\leq \delta , \end{array}$$ (M2)

where ǁY_i_ǁ_0_ is the L_0_-norm (aka the cardinality) of Y_i_, denotes the noise level, and _i_ > 0 is some appropriately chosen weighting parameter. The corresponding convex relaxation model is:

$$\begin{array}{c} \min{\left\|X\right\|}_{*}+{\Sigma_{i=1}}^{K}\rho_{i}{\left\|Y_{i}\right\|}_{1}\\ s.t.\;X+Y_{i}+Z_{i}=M_{i},\;i=1,\ldots ,K\\ {\left\|Z\right\|}_{F}\leq \delta . \end{array}$$ (M3)

Note that (M3) becomes a common-vector model (M1), when we add an additional constraint X = x* ι to it.

Refer to the following for the pseudo code for the common-background and sparse-foreground decomposition model (M1).

Input: The data matrix *M*, and the noise level parameter δ.

Output: The common-background vector *x* and the sparse-foreground matrix*Y*.

Begin:

(Initialization). Define the augmented Lagrangian function for (M1):

$$L\left(x,Y,Z;D\right)≔{\left\|Y\right\|}_{1}-\langle D,x^{*}I+Y+Z-M\rangle +\frac{\gamma }{2}{\left\|x^{*}I+Y+Z-M\right\|}_{F}^{2}$$

Note that *D* is the Lagrange multiplier associated with the equality constraint in (M1), and *r* > 0 is a penalty parameter. Set initial values: *Y*: = *Y* ^0^, *Z*: = *Z* ^0^, *D*; = *D* ^0^. Set value for parameter *r*. Set the loop counter *k*: = 0.

(Minimizing the augmented Lagrangian function with respect to *x*, *Y*, *Z* alternatingly). Solve the following three simple optimization problems sequentially:

$$\begin{array}{c} x^{k+1}≔argmin_{x}L\left(x,Y^{k},Z^{k};D^{k}\right)\\ Y^{k+1}≔argmin_{Y}L\left(x^{k+1},Y,Z^{k};D^{k}\right)\\ Z^{k+1}≔argmin_{\left\|Z\right\|{}_{F}\leq \delta }L\left(x^{k+1},Y^{k+1},Z;D^{k}\right) \end{array}$$

(Updating the Lagrange multiplier). Compute

$$D^{k+1}≔D^{k}-\gamma \left(x^{k+1}*I+Y^{k+1}+Z^{k+1}-M\right)$$

(Stopping criterion). If certain stopping criterion is met, then stop. Otherwise, set *k*: = *k* +1, and go to Step 1.

(Outputting *x* and *Y*). Output the common-background vector *x* ^*k+1*^ and the sparse-foreground matrix *Y* ^*k+1*^.

### The Maximum Block Improvement (MBI) co-clustering module

Our clustering approach is based on a tensor optimization model and an optimization method termed Maximum Block Improvement (MBI) [29]. Consider the following formulation for the co-clustering problem for a given tensor data set M ∈ R^n1×n2 … ×nd^:

$$\begin{array}{cc} & \min{\Sigma_{j1=1}}^{n1}{\Sigma_{j2=1}}^{n2}\ldots {\Sigma_{jd=1}}^{\mathrm{nd}}f\left(M_{j1,j2,\ldots ,\mathrm{jd}}-{\left(X\;x_{1}\;Y^{1}\;x_{2}\;Y^{2}\;x_{3}\ldots x_{d}\;Y^{d}\right)}_{j1,j2,\ldots ,\mathrm{jd}}\right)\\ & s.t.\;X\in R^{p1\times p2\ldots \times \mathrm{pd}},Y^{j}\in R^{\mathrm{nj}\times \mathrm{pj}}\;\text{is a row assignment matrix},\;j=1,2,\;\ldots ,d, \end{array}$$

where f is a given proximity measure. In [29], the so-called *Maximum Block Improvement* (MBI) method is proposed to solve the above model (CC), with encouraging numerical results. Interested readers are referred to our previous work in [29] for the pseudo-codes of the MBI model for tensor co-clustering and for 2D matrix co-clustering. Note that the above model for tensor co-clustering is *exact*, in the sense that if exact co-clusters exist then the above model at its optimum achieves the minimum value zero.

The MBI clustering approach can be applied to co-cluster gene expression data in 2D matrices (genes versus samples) as well as data in high-dimensional tensor form. The new framework is flexible in that it is easy to incorporate a variety of clustering quality measurements. Our preliminary experimental testing demonstrates its efficiency and effectiveness [30, 29]. MBI, as a checkerboard co-clustering approach, without any gene-trimming, could provide identification of cancer subtypes and also genes correlated with the subtypes at the same time, while most previous bi-clustering or co-clustering approaches (e.g. LAS [31], QUIBC [32], etc) are more focused on extracting coherent gene expression patterns, usually not perform well for cancer subtyping. Theoretically, compared to other co-clustering approaches, our model is based on an exact formulation for co-clustering while searching for an approximate solution for the exact model. In this vein, other approaches (e.g. the SVD low-rank matrix method [33] and the NMF method [17]) base the efforts on an approximate formulation of co-clustering.

Take the NMF method as an example, which is one of the currently widely-used approaches for cancer molecular subtyping. There are two inherent shortcomings for NMF: (1) it requires the entries of the input gene expression matrix to be all non-negative values; (2) it divides the input matrix into the same number of groups for the rows (genes) and for the columns (samples). Since the number of the genes (~30,000) is usually significantly greater than the number of the samples (about several hundreds), it may not be very meaningful to divide the genes (rows) and the samples (columns) into the same number of groups, where usually the number of different molecular subtypes is small, say between 2 and 5. For example, when the number of groups k = 2, the NMF method will get a 2×2 separation of a lager gene expression matrix (such as 22,000 rows × 276 columns) into 4 blocks, yielding a very rough separation of the matrix. On the same footing our MBI approach is flexible enough to yield a properly fine-detailed separation, say, with the number of row groups k_1_>100 and the number of column groups k_2_ = 2.

We would like to point out that the numbers of k_1_ and k_2_ are important dimension parameters for MBI co-clustering. There are no efficient methods that could derive the optimal numbers for k_1_, k_2_, but we could apply a local search process [29] to search for a local optimal numbers for k_1_, k_2_.

Note that almost all unsupervised clustering approaches will not always generate exactly the same clusters form all the runs with different parameter setups on the same dataset. Like the NMF approach, the new MBI algorithm may or may not converge to the same solution for each run, depending on the different random initial conditions. We also apply the idea of consensus clustering, taking into account the information of every two samples being clustered together from a certain number of MBI runs. If two samples are of the same type or subtype, we then expect that sample assignments vary little from run to run [17].

### Novel features of our new framework SPARCoC

**Fig 2:**
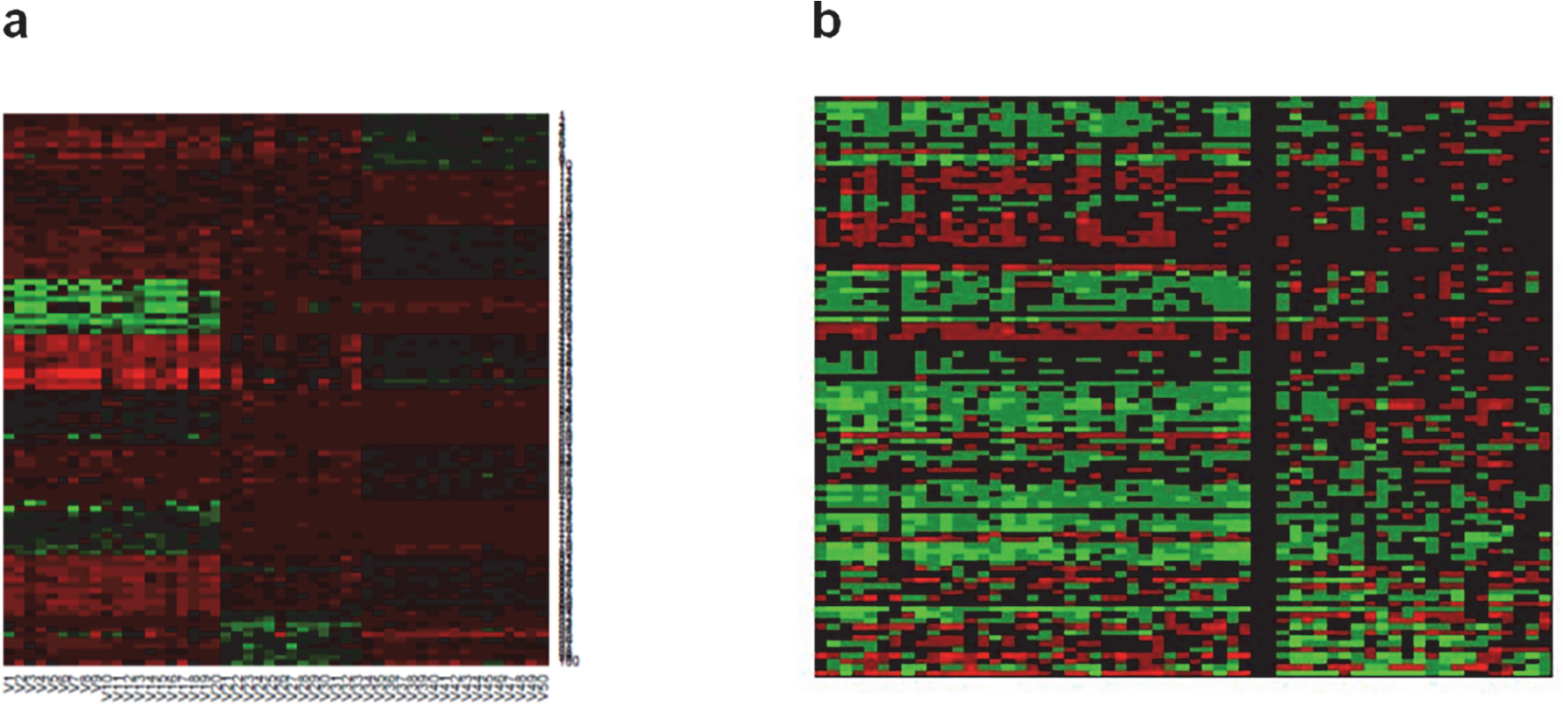
Heat maps clearly show the patterns of MBI co-clusters . For the gene expression datasets studied here, MBI co-clustering simultaneously provide the gene (row) groupings and the sample (column) groupings, identifying the genes associated with the different types or subtypes. **(a)** Heat map shows clear co-clusters identified by MBI. The plot is based on real values of Y matrix of gene expression profiling data (data1 with three types: COID/20, CM/13, NL/17; refer to S1 File). Each row corresponds to one gene; each column corresponds to one sample. This heat map shows the expression values of 100 genes across all the 3 different types. **(b)** Heat map shows clear co-clusters identified by MBI. The plot is based on the values of Y matrix for Canada stage1 dataset (heat map for Canada stage1 dataset with 562 genes with k_1_ = 100 and k_2_ = 2. The two groups are separated by a thick black vertical line).

The following provides the fundamental of the Common-background and Sparse-foreground Decomposition (CSD) model and the Maximum Block Improvement (MBI) co-clustering technique, and also summarizes briefly the novel features of SPARCoC compared with existing clustering methods:

- Where are the cancer genes important for defining different molecular subtypes of cancer? One of the major discoveries through our study indicates that they represent the “foreground” of the gene expression profiling data of patients, typically hidden within the “background” of an ocean of noisy gene expression data. The effort of our new clustering framework based on CSD decomposition and MBI co-clustering is to define distinct molecular subgroups of patients and to help single out the important impact-making “foreground” genes from their noisy background. *Note that almost all other current clustering and co-clustering methods are based on the notion of identifying the commonality; hence they are trapped by the patterns of the background*, *rather than focusing on the information-rich “foreground” of the gene expression data* (see **Fig. 1A**).
- The CSD decomposition module facilitates the effect of the important “interesting” genes to stand out of the “background”, thus help identify cancer genes and fine-detailed molecular subtypes, which will otherwise be impossible to detect (see **1A**, **Table 1**).
- The MBI co-clustering module, as a checkerboard co-clustering approach, can generate both row grouping and column grouping at the same time, and thus help identify cancer genes (rows) defining the different molecular clusters/subgroups of patients (columns) (see **Fig. 2**).
- Our approach can be applied to large scale genomic profiling datasets of patients without any gene trimming or feature selection. It turns out to be very efficient and runs on whole-genome gene expression datasets as well as other datasets such as mutation, copy number, miRNA, methylation, exome sequencing and reverse phrase protein array etc. It is able to identify potentially new molecular subtypes of cancer and cancer genes or gene patterns.

**Table 1:** Performance of NMF (with k = 2 sample clusters) on original matrices Ms or normalized matrices L, compared with its performance on CSD decomposed matrices M_Y or L_Y.

Datasets	NMF runs (each dataset with 10 runs)	p-value (5-year overall survival)	Statistically valid (1: p-value <0.05; 0: otherwise)
**ACC_stage1_original_M**	1	0.0483	1
	2	0.0483	1
	3	0.0483	1
	4	0.0483	1
	5	0.0483	1
	6	0.0483	1
	7	0.0483	1
	8	0.0483	1
	9	0.0483	1
	10	0.0483	1
**ACC_stage1_M_Y *(from CSD decomposition)***	1	0.0195	1
	2	0.0302	1
	3	0.0447	1
	4	0.0446	1
	5	0.0196	1
	6	0.0447	1
	7	0.0447	1
	8	0.0447	1
	9	0.0301	1
	10	0.0195	1
**Jacob_stage1_original_M**	1	0.5361	0
	2	0.5361	0
	3	0.5361	0
	4	0.5361	0
	5	0.5361	0
	6	0.5361	0
	7	0.5361	0
	8	0.5361	0
	9	0.5361	0
	10	0.5361	0
**Jacob_stage1_normalized_L**	1	0.1201	0
	2	0.1326	0
	3	0.1121	0
	4	0.1135	0
	5	0.1381	0
	6	0.1201	0
	7	0.1201	0
	8	0.1326	0
	9	0.1291	0
	10	0.1201	0
**Jacob_stage1_L_Y *(from CSD decomposition)***	1	0.0019	1
	2	0.0041	1
	3	0.0061	1
	4	0.0031	1
	5	0.0041	1
	6	0.0031	1
	7	0.0031	1
	8	0.0052	1
	9	0.0019	1
	10	0.0041	1

Note that since NMF uses random seed approaches, 10 runs were performed for each data set and the one with the lowest p-value (by survival log-rank analysis) was highlighted. From testing on the ACC stage1 dataset, the performance of NMF clustering is better (i.e., smaller p-values) on M_Y than on M, where M is the original Jacob gene expression matrix, and M_Y is the sparse matrix from CSD decomposition. From testing on the Jacob stage1 dataset, the performance of NMF clustering is much better on L_Y than on L or on M, where M is the original Jacob gene expression matrix, L is the normalized matrix, and L_Y is the sparse matrix from CSD decomposition. ***NMF could not get statistically-significant OS different separations of the Jacob stage1 samples using the original M matrix or the normalized L matrix*, *but it could do so using the sparse matrix L_Y from CSD decomposition***.

**Fig 3:**
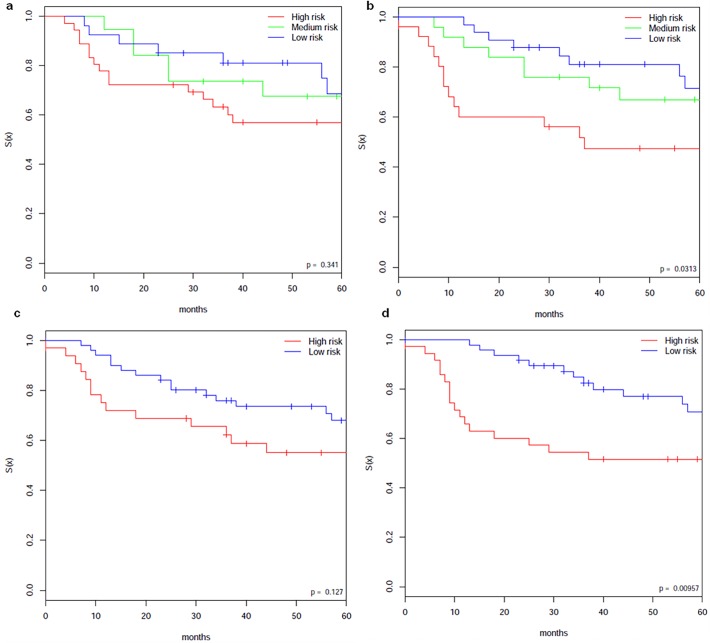
Comparison of the cluster of Hierarchical clustering (Hclust) versus that of MBI, and the cluster of NMF versus that of MBI. (a) and (b). Comparison of Kaplan-Meier survival plots based on the unsupervised clusters of Hierarchical clustering (Hclust) and that of MBI, when given the same gene expression matrix M (lung ADCA Canada dataset from Shedden et al. [7]. (a) Kaplan-Meier survival plot based on Hclust. (b) Kaplan-Meier survival plot based on MBI clustering (with leave-one-out-cross-validation (LOOCV) ~99% accuracy). MBI shows a better separation of the aggressive subgroup from the other two subgroups compared with the Hclust Bryant et al. [6]. The p-values are calculated by log-rank test; The LOOCV was done using PAM [18]. (c) and (d). Comparison of Kaplan-Meier survival plots based on the unsupervised clustering of NMF (c) and that of MBI (d), when given the same gene expression matrix M (lung ADCA Canada dataset from Shedden et al. [7]). When given the same gene expression testing data, the survival curves from MBI clustering shows a more significant separation than those from NMF clustering. The p-values are calculated by log-rank test.

Refer to the testing results provided here and in the Supporting Information (*see* ***S1 File*** *for additional testing results*), which demonstrate the clear advantages of our new clustering framework. Our testing results show that: (1) the CSD approach facilitates the identification of gene markers, making potential gene markers stand out of the “background”; (2) the MBI approach performs better on Y versus on M, where M is the original gene expression matrix and Y is the sparse matrix generated through CSD decomposition; (3) our new clustering framework performs much better in comparison with the widely used clustering approaches, e.g., Hclust and NMF (also see **Fig. 3A and 3B, Fig. 3C and 3D;** the smaller p-values from log rank test (**Fig. 3; Table 2**) and the lower percentages of 3-year overall survival of high-risk groups (*also see* ***S1 File*** *for additional testing results*) implicate our CSD+MBI model is a better clustering model).

**Table 2:** Performance comparison of NMF (k = 2, i.e., 2 sample clusters) and MBI (k_1_ = 100, k_2_ = 2) on different stage I lung ADCA datasets.

Datasets	Clusters of NMF		Clusters of MBI	
	p-value (5-year overall survival)	Statistically valid (1: p-value <0.05; 0: otherwise)	p-value (5-year overall survival)	Statistically valid (1: p-value <0.05; 0: otherwise)
**ACC_stage1_original_M**	0.0483	1	0.0195	1
**ACC_stage1_M_Y *(from CSD decomposition*)**	0.0195	1	0.0164	1
**Jacob_stage1_original_M**	0.5361	0	0.0045	1
**Jacob_stage1_normalized_L**	0.1201	0	0.0011	1
**Jacob_stage1_L_Y *(from CSD decomposition)***	0.0019	1	0.0018	1

Note that since NMF and MBI use random seed approaches, 10 runs were performed for each data set and the one with the lowest p-value (by survival log-rank analysis) from the 10 runs was selected. **Compared with the performance of NMF, the performance of MBI is more robust; When both achieve statistically valid clusters, MBI clusters have smaller p-values from log rank test, which implicates MBI is a better clustering model**.

Compared with other unsupervised clustering methods, our new clustering framework performs robustly overall, and demonstrates a substantially improved clustering result on certain datasets. Indeed the performance of a clustering algorithm may be significantly affected by the datasets: some datasets with distinct types as “apple and orange” types, while some other datasets with types having very subtle difference as different “apple” types. The aim of this paper is in fact to propose a carefully designed new effective clustering framework, in order to meet the challenges in cancer heterogeneous molecular subtyping (differentiating subtly altered “apple” types). In the following, we apply our new framework to study the very challenging, extreme heterogeneous lung cancer adenocarcinoma (lung ADCA and stage I lung ADCA).

## Results

In this section we have analyzed high-quality gene expression profiling data of collectively ~600 patient samples, and our method readily provides clusters of lung ADCA patients with distinct clinical survival outcomes and identifies gene signatures, which, when verified using completely independent datasets, are able to distinguish lung ADCA patients into subgroups with significantly different overall survival (p-values < 0.05). We could replicate our findings using completely independent datasets. Statistical analyses are conducted to demonstrate robustness of the results.

We use SPARCoC to analyze gene expression profiles of lung adenocarcinoma (ADCA) patients and present our results of molecular subtyping and prognostic gene signature discovery. Based on whole-genome gene expression profiling of lung ADCA patients, SPARCoC clusters the patients into distinct subgroups; and patient overall survival is significantly different among the subgroups. It helps identify cancer gene signatures, which, when verified with completely independent gene expression profiling data, could separate lung ADCA and stage I lung ADCA patients into subgroups with different clinical survival outcomes. *Note that the results presented here are based on the gene expression profiling data analysis only, without incorporating any other feature selection, or clinical information, which is different from other analysis in the literature (e.g., [34, 35, 15]). However, still we can see that we are able to replicate our findings with completely independent datasets*.

For testing and verification, we use in our study the following datasets with gene expression profiles of collectively more than 600 lung ADCA patient samples; these large datasets are of high quality and are collected with extensive clinical information of the cancer patients.

### Datasets used

#### Jacob dataset

442 ADCA samples, with gene expression and clinical data from the National Cancer Institute (NCI) Director’s Challenge Consortium [11]. This dataset consists of 4 different patient cohorts, including Toronto/Canada (TC, n = 82, with stage I n = 57), Memorial Sloan-Kettering Cancer Center (MSKCC, n = 104, with stage I n = 62), H. Lee Moffit Cancer Center (HLM, n = 79, with stage I n = 41), and University of Michigan Cancer Center (UM, n = 177, with stage I n = 116). Similar as in [15], datasets TC and MSKCC are combined together called TM (n = 186), and datasets HLM and UM combined together called HM (n = 256).

#### ACC dataset

117 ADCA samples of Aichi Cancer Center, obtained from http://www.ncbi.nlm.nih.gov/geo, accession number GSE13213 [36].

#### GSE5843 dataset

46 ADCA samples (stage IA 16 samples; stage IB 30 samples), obtained from http://www.ncbi.nlm.nih.gov/geo, accession number GSE5843 [37].

It is known that lung cancer is the leading cause of cancer-related death worldwide (http://seer.cancer.gov/statfacts/). Nearly 50% of patients with stages I and II non-small cell lung cancer (NSCLC) eventually die from recurrent disease despite surgical resection. It is meaningful to discover lung cancer molecular subtypes with distinct clinical outcomes such that each molecular subtype has proposed treatment guidelines that include specific assays, targeted therapies, and clinical trials. However, it is difficult to study the subtle heterogeneous differences of molecular subtypes of lung adenocarcinoma (ADCA) and especially those of stage I lung ADCA, without access to clusters from powerful unsupervised clustering approaches such as the novel clustering framework SPARCoC developed here (refer to the performance comparison of our clustering approach and NMF or Hclust in the previous section and S1 File).

### Clustering lung adenocarcinoma (ADCA) patients

#### Distinct subgroups of patients of TM and HM datasets

**Fig 4:**
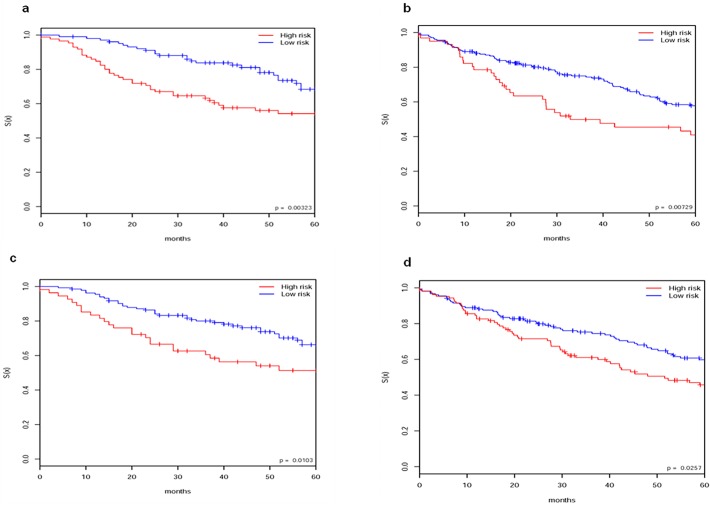
Consistent TM clusters and consistent HM clusters . (a) TM clusters and (b) HM clusters. Kaplan-Meier plots of the two consistent TM clusters and the two consistent HM clusters from our clustering approach with CSD (decomposition noise level is 20,000) and MBI (10 runs with parameter k_2_ = 2). (c) Sample classification of TM samples using the 128 genes, based on the subtypes of HM, and (d) Sample classification of HM samples using the 128 genes, based on the subtypes of TM. TM and HM cross-validation (CV) using 128 genes (where 128 genes are identified from all genes based on clusters of MBI 10 runs on TM and MBI 10 runs on HM; For cross-validation, least square based sample prediction is applied).

The TM and HM datasets were used as the training datasets for our analysis. We first applied our MBI clustering approach to the decomposed TM and HM data matrices from CSD and obtained consistent clusters for TM and HM samples respectively. Kaplan-Meier plots showed statistically significant differences in overall survival (OS) (p-values: p = 0.00323 for TM and p = 0.0106 for HM by log-rank test) between the two clusters of patients for each dataset (**Fig. 4A and Fig. 4B**). The results of the leave-one-out-cross-verification (LOOCV) of the two clusters of TM and HM are 0.96 and 0.80, respectively.

#### Identification and independent verification of cancer genes as prognostic gene signature

Based on the clusters of TM and those of HM, t-test (p-value cutoff of 0.01) was applied to identify genes that are differentially expressed in both datasets, and we got 1945 genes. From these genes we then selected genes based on the information of the Network of Cancer Genes (NCG 3.0) [http://bio.ieo.eu/ncg3/index.html]. We identified 128 genes from the analysis of TM and HM clusters. Refer to the **S1 File** (http://bioinformatics.astate.edu/code) for the 128 genes and related pathway information.

We conducted TM and HM cross-validation (CV) using the 128 genes. That is, we used the identified two TM clusters to conduct prediction for each of the HM samples and assign the HM samples into two clusters. Similarly, we used the identified two HM clusters to conduct prediction for each of the TM samples and assign the TM samples into two clusters. For sample prediction we applied the scoring function to minimize the least square. The cross-validation could separate the patients into two consistent clusters for TM and HM respectively. Kaplan-Meier plots showed statistically significant differences in OS (p = 0.00666 for TM and p = 0.0157 for HM by log-rank test) between the two predicted clusters of patients for each dataset (**Fig. 4C and Fig. 4D**).

**Fig 5:**
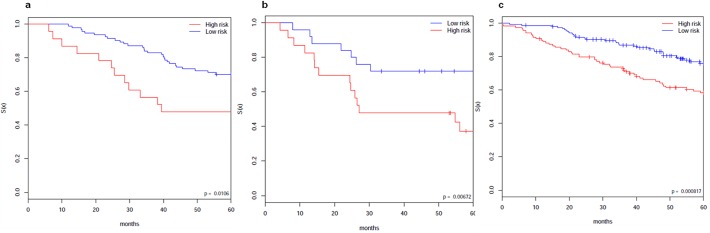
Independent verification testing of the 128 identified genes. Testing of the 128 identified genes on the ACC (a) and the GSE5843 dataset (b). Kaplan-Meier plots of the clusters of the samples show statistically significant survival differences, with p-value = 0.0106 for the ACC dataset, and p-value = 0.00672 for the GSE5843 dataset. For each verification test, the separation of the samples is from MBI running with parameter k_2_ = 2 on the corresponding rows of the dataset (i.e., using only the part of Y matrix of ACC or GSE5843 that corresponds to the 128 genes). (c) Testing of the 128 identified genes on Jacob stage1. The separation of the samples is from MBI running on the corresponding rows of the dataset with parameter k_2_ = 2. Kaplan-Meier plot of the sample clusters show statistically significant survival differences, p-value = 0.000817.

For independent verification, we used the ACC dataset. We mapped the 128 genes by gene symbol to the ACC dataset and then ran our clustering approach using only the mapped genes. From the verification, we got consistent clusters for the ACC dataset. Kaplan-Meier plots showed statistically significant differences in OS (p = 0.0106 by log-rank test) between the 2 subgroups of patients (**Fig. 5A**).

Similarly for another independent verification, we used the GSE5843 dataset. We mapped the 128 genes by gene symbol to the GSE5843 dataset and then ran our clustering approach using only the mapped genes. From the verification, we got consistent clusters for the dataset. Kaplan-Meier plots showed statistically significant differences in OS (p = 0.00672 by log-rank test) between the 2 subgroups of patients (**Fig. 5B**).

Also, we tested the 128 genes on the stage I of the Jacob dataset. Through applying our clustering approach to the 128 genes/rows of the dataset, we also got consistent clusters for Jacob stage1. Kaplan-Meier plot showed statistically significant differences in five year survival (with p = 0.000617, by log-rank test) between the 2 clusters of patients for the dataset (**Fig. 5C**).

### Clustering stage I lung adenocarcinoma (ADCA) patients

Clear separation of stage I lung ADCA patients into aggressive and non-aggressive groups is difficult [38, 35]. There are very few robust gene signatures published in the literature for defining the high-risk and low-risk groups of stage I lung ADCA. As in the previous section, we conducted a similar analysis for stage I lung ADCA through applying our new framework.

#### Distinct subgroups of patients of ACCstage1 and Jacobstage1 datasets

**Fig 6:**
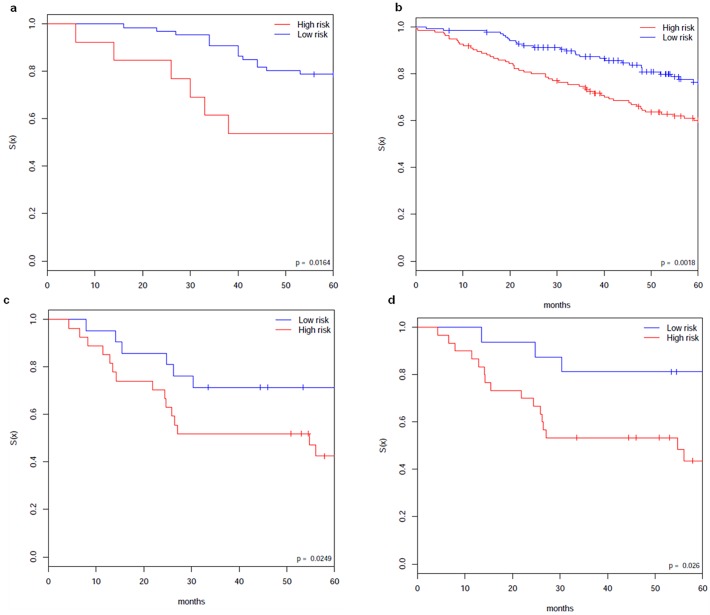
Clustering of stage I samples. (a) and (b). Kaplan-Meier plots of the consistent clustering of ACC stage1 (a) and that of Jacob stage1 (b) from our clustering approach. The clusters identified by our clustering approach show statistically significant survival differences. (c) and (d). Comparison of the sample separation based on the 144 identified genes and the separation based on the stage information of the GSE5843 dataset. (c) Independent verification testing of the 144 identified genes on GSE5843. Kaplan-Meier plots of the clusters of the samples, which shows statistically significant survival differences. The clusters is from MBI running with parameter k_2_ = 2 on the corresponding rows of the dataset (i.e., using only the part of Y matrix of GSE5843 that corresponds to the 144 genes). p-value = 0.0249. (d) Kaplan-Meier plots of the clusters of the samples based on the separation of stage IA and IB. p-value = 0.026.

For discovery we used the datasets of ACCstage1 and Jacobstage1 as the training datasets. We first applied our clustering approach to the datasets and got a consistent clustering for each dataset respectively. Kaplan-Meier plots showed significant differences in OS (p = 0.0164 for ACCstage1 and p = 0.0018 for Jacobstage1 by log-rank test) between the 2 clusters of patients for each dataset (**Fig. 6A and Fig. 6B**).

#### Identification and independent verification of cancer genes as prognostic gene signature of stage I lung ADCA

Based on the clustering of the datasets, t-test (p-value cutoff threshold of 0.01) was applied to identify differentially expressed genes. We found 144 common genes from the datasets of ACCstage1 and Jacobstage1. The identified 144 genes are differently expressed between high-risk and low-risk groups. Note that we do not know how they are compared with their gene expression of normal lung, which may be further explored with additional datasets. Refer to the **S1 File** (http://bioinformatics.astate.edu/code) for the 144 genes and related pathway information.

For independent verification of the 144 identified genes, we used the GSE5843 dataset. We mapped the 144 genes by gene symbol to the GSE5843 dataset and then run our clustering approach using only the mapped genes. From the verification, we got consistent clusters for the dataset. Kaplan-Meier plots showed statistically significant differences in OS (p = 0.0107 by log-rank test) between the 2 subgroups of patients. Compared with the clusters of the samples based on the separation of stage IA and IB, the smaller p-value shows that the separation based on the 144 genes is meaningful (**Fig. 6C and Fig. 6D**).

## Discussion

In summary, we presented a new unsupervised clustering framework SPARCoC (Sparse-CoClust) for molecular pattern discovery and cancer gene identification. We applied the framework to study the extreme heterogeneous lung ADAC molecular subtyping and gene signature discovery for predicting survival outcomes of patients. Compared to other currently widely-used clustering methods for cancer molecular subtyping study, such as Hclust and NMF, our new framework has demonstrated clear advantages. SPARCoC has the abilities to facilitate cancer molecular subtyping, cancer gene identification through the CSD foreground detection and the MBI co-clustering, from the genome-wide noisy gene expression “background”.

Note that the CSD is a novel decomposition model which is different from the so-called RPCA model proposed in the literature; this is the first time that such a model has been applied to gene expression analysis for common-background and sparse-foreground decomposition, and has clearly demonstrated its advantages. Also note that the MBI model for tensor co-cluster is exact, in the sense that if exact co-clusters exist then the model at its optimum achieves the minimum value zero. Among almost all the current co-clustering methods in the literature for gene expression data analysis, some kind of approximations are always present in the modeling part. For instance, the SVD or PCA approaches are based on the observation that the important information regarding the bi-clusters should be present in the vectors (eigenvectors representing the principal singular values). Notwithstanding their insights, these methods are heuristic in nature. The same can be said about the NMF approach. Among the methods we studied in the literature, the only two exceptions are the model in [39] and our MBI model [29], where the co-clustering models are exact although approximation algorithms are applied to solve the exact models. Among the models in [39] and [29], our MBI is generally designed for co-clustering for multi-dimensional tensor datasets, and there is a convergence assurance for the algorithm. We have reasons to believe that the MBI model is robust in the settings of the parameters, while the final solution it provides may still be dependent on the parameter settings as well as the preprocessing of the datasets for molecular subtyping study.

Through our new framework SPARCoC, we identified consistent clusters of lung cancer patients with statistically significant survival differences and new gene signatures for lung ADCA and stage I lung ADCA. The identified gene signatures could define distinct subgroups of lung ADCA or stage I lung ADCA patients with different clinical survival outcomes. Especially for stage I lung ADCA, there are very few gene signatures that have been developed in the literature. We have identified gene signatures from the training dataset and then verified them through completely independent available datasets. Further tests and verifications will be conducted using newly released genomic profiling data of lung cancer patients (e.g., data from TCGA: http://cancergenome.nih.gov/). Relevant wet-lab testing and verification of new gene signatures are needed for potential direct medical applications. Also note that recent studies from different labs have also shown that lung cancer molecular intrinsic subtypes involve difference in genome-wide gene expression and pathways, and other alterations in patient tumors [12, 14]. Therefore, our future study of molecular subtyping of lung ADCA will integrate our clustering framework with other molecular information, protein-protein interaction (PPI), gene regulatory network (GRN), transcription factor (TF), etc.

The new framework SPARCoC is capable of handling high-dimensional (e.g., 2D of gene versus samples, and 3D or 4D of gene versus samples versus time-points versus tissues [29, 30]) and large scale integrated genomic data. It is very flexible in nature: it can be applied not only to whole-genome mRNA gene expression data, but also to other 2D or even high-dimensional molecular information, such as DNA methylation chips, single nucleotide polymorphism (SNP) arrays, microRNA sequencing, and reverse-phase protein arrays (RRPA) etc. SPARCoC is a powerful framework for clinically and biologically meaningful pattern discoveries, which will empower studies of molecular subtypes of cancer and cancer gene identification.

Note: Supporting S1 File is available for the paper.

## Supporting Information

S1 File Combined file of supporting figures and tables. Table S1. Performance comparison of our approach vs the approach NMF on datasets of different lung cancer histological types. Table S2. Comparison of 3-year overall survival (%) of the patient grouping of different clustering methods. Fig. S1. Comparison of the clusters in the literature and the clusters generated by MBI. Fig. S2. Comparison of sample grouping using matrix M vs matrix Y of the ACCstage1 dataset. Fig. S3. MBI helps identify “noise or outlier” sample from the gene expression dataset.
(DOCX)
