# Supplementary material for: SPARCoC: A New Framework for Molecular Pattern Discovery and Cancer Gene Identification
Source: PLoS One. 2015 Mar 13;10(3):e0117135. doi: 10.1371/journal.pone.0117135 (PMC4359112; doi:10.1371/journal.pone.0117135)
Supplement: S1 File — Table S1. Performance comparison of our approach vs the approach NMF on datasets of different lung cancer histological types. Table S2. Comparison of 3-year overall survival (%) of the patient grouping of different clustering methods. Fig. S1. Comparison of the clusters in the literature and the clusters generated by MBI. Fig. S2. Comparison of sample grouping using matrix M vs matrix Y of the ACCstage1 dataset. Fig. S3. MBI helps identify “noise or outlier” sample from the gene expression dataset. (DOCX) [file pone.0117135.s001.docx]

SUPPLEMENTARY INFORMATION FOR THE PAPER:

**SPARCoC: a new framework for molecular pattern discovery and cancer gene identification**

**Datasets.** The following are the histological lung cancer datasets and the lung adenocarcinoma (ADCA) datasets we used in our study. For detailed information of each dataset, please refer to the corresponding reference papers.

**Terminology and abbreviations:**

Lung cancer; normal lung (NL); small cell lung cancer (SCLC); non-small cell lung cancer (NSCLC); squamous cell carcinoma (SQ); adenocarcinoma (ADCA); large cell carcinoma (LC);

Common-background and Sparse-foreground Decomposition (CSD decomposition);

Co-clustering based on Maximum Block Improvement (MBI co-clustering)

**Dataset description.**

**data-1: NL/17, COID/20, CM/13; SCLC/6 (normalized from 1b)**

12600×56; Among the 56 samples, Columns 1~20: pulmonary carcinoid samples (Carcinoid); Columns 21~33: colon cancer metastasis samples (Colon); Columns 34~50: normal lung samples (Normal); Columns 51~56 (not used here): small cell carcinoma samples (SmallCell) [1-3].

**data-2: ADCA/127, SQ/21, NL/17, COID/20, CM/12, SCLC/6;**

**(Setup1: NL/17, COID/20, CM/12; setup2: SQ/21, NL/17, COID/20, CM/12; setup3: ADCA/127, SQ/21)**

 12600×186; The original dataset includes a total of 203 snap-frozen lung tumors (186 samples) and normal lung (17 samples) specimens. There are 186 samples of the lung cancer dataset (127 samples of histologically defined lung adenocarcinomas, 21 samples of squamous cell lung carcinomas, 20 samples of pulmonary carcinoids and 6 samples of small cell lung carcinomas (SCLC), 12 samples of other adenocarcinomas which were suspected to be extrapulmonary metastases based on clinical history). For detailed description of the dataset, refer to [3] and its supporting information.

**ADCA and ADCA stage1**:

**Wilkerson DataSet**: 116 adenocarcinomas (ADCA) samples, three subtypes: bronchoid: 1-47 (n=47) samples; squamoid: 48-76 (n=29) samples; magnoid: 77-116 (n=40) samples, based on Wilkerson’s classification [4].

**Jacob Dataset**: 442 adenocarcinomas (ADCA) samples, with gene expression and clinical data from the National Cancer Institute (NCI) Director’s Challenge Consortium [5]. This data set consisted of 4 different patient cohorts, including Toronto/Canada (TC, n=82), Memorial Sloan-Kettering Cancer Center (MSKCC, n=104), H. Lee Moffit Cancer Center (HLM, n=79), and University of Michigan Cancer Center (UM, n=177).

**ACC Dataset**: 117 ADCA samples from Aichi Cancer Center, obtained from http://www.ncbi.nlm.nih.gov/geo, accession number GSE13213 [6].

**Table S1. Performance comparison of our approach vs the approach NMF on datasets of different lung cancer histological types**. We test the performance of MBI on Y (Y matrix from CSD decomposition) data vs. NMF on M (transformed M matrix with no negative values) data. Since both MBI and NMF use random seed approaches, ten runs were performed for each data set and the one with the best performance was chosen. Parameter setup for both MBI and NMF was adjusted according to the number of known groups. The predicted groups of all the samples were then compared with the known data. **From the testing it is clear that both our approach and the approach NMF could 100% correctly cluster the samples of data-1, however the performance of our approach is significantly better than NMF when tested on the other datasets of different lung cancer histological types.**

| **Datasets with different lung cancer histological types** | **No. of genes × No. of total samples;**  **Types (hist. types/#samples** | **CSD+MBI** | | **NMF** | |
| --- | --- | --- | --- | --- | --- |
|  |  | **No. of correctly clustered samples/all testing samples** | ***Percentage of correctly clustered samples*** | **No. of correctly clustered samples/all testing samples** | ***Percentage of correctly clustered samples*** |
| data-1 | 12600×50; 3  (NL/17, COID/20, CM/13) | 50/50 | **100%** | 50/50 | **100%** |
| data-2 setup1 | 12600×49; 3  (NL/17, COID/20, CM/12) | 47/49 | **96%** | 31/49 | **63%** |
| data-2 setup2 | 12600×70; 4  (SQ/21, NL/17, COID/20, CM/12) | 65/70 | **93%** | 51/70 | **73%** |
| data-2 setup3 | 12600×148;2 (ADCA/127, SQ/21) | 97/148 | **66%** | 84/148 | **57%** |

**Table S2. Comparison of 3-year overall survival (%) of the patient grouping of different clustering methods.** The same dataset is used for these comparison testing; with slightly different no. of samples for each test. **Compared with the 3-year overall survival rates of the patient subgroups identified from the other methods, our approach identified low-risk subgroups of patients showing higher rates of 3-year overall survival, and high-risk subgroups of patients showing lower rates of 3-year overall survival.**

| **Different clustering methods** | ***Predicted low-risk group (%)*** | ***Predicted high-risk group (%)*** | No. of samples of  predicted low-risk group / high-risk group | Survival Plot Figures |
| --- | --- | --- | --- | --- |
| NMF | 66.7 | 58.0 | 51/31 | see Fig. 3(c) |
| **MBI (k_2_=2)** | **70.8** | **55.9** | 48/34 | **see Fig. 3(d)** |
| Hclust | 67.9 | 56.8 | 28/37 | see Fig. 3(a) |
| **MBI (k_2_=3)** | **72.0** | **48.0** | 25/25 | **see Fig. 3(b)** |
| Stage (Stage I vs II) | 69.6 | 50.0 | 56/26 |  |

**Testing results demonstrate the advantage of MBI compared with other widely-used clustering approaches: hierarchical clustering (Hclust) or Consensus clustering.** MBI performs better than Hclust and consensus clustering on ADCA clustering in the literature.

|   a |  b |
| --- | --- |
|   c |   d |

**Figure S1**. **Comparison of the clusters in the literature and the clusters generated by MBI.** **(a) and (b) comparison on CanadaDFCI 82 ADCA samples.** We have conducted the comparison of MBI co-clustering with the Hierarchical clustering of Bryant et al. [7] for 82 ADCA samples, the two groupings are about 49/82 = 60% consistent). Here are the Kaplan-Meier survival plots on **Bryant clustering (a)** and based on **MBI clustering (b)** (Leave-one-out-cross-validation (LOOCV) is ~99% accuracy, missed 1 out of the 89). MBI shows a good separation for the ADCA aggressive subgroup from the other two subgroups. **(c) and (d) comparison On Wilkerson dataset of 116 ADCA samples.** We compare MBI co-clustering grouping with the ConsensusClusterPlus of Wilkerson et al. [8] for the 116 ADCA samples. MBI 3 groups of the 116 samples got 100% correct with leave one out cross validation (LOOCV). The Wilkerson's 3 groups of the 116 samples got 105 out of 116, or 90.5% correct with LOOCV. The survival curves based on **Wilkerson grouping (c)** and **MBI grouping (d)**; MBI shows a good separation for the three ADCA subgroups. Note that the LOOCV was done using PAM [9].

**MBI on Y vs M: MBI running on Y gets improved ADCA sample clustering**.

|  |  |
| --- | --- |

1. (b)

**Figure S2**. **Comparison of sample grouping using matrix M vs matrix Y of the ACCstage1 dataset.** (a) Survival image of ACCStage1, M matrix. (b) Survival image of ACCStage1, noise10 Y matrix. We can see from the two survival images the subtle difference of the grouping generated by Y and the grouping generated by M. This shows the improved sample grouping using matrix Y instead of matrix M of the ACCstage1 dataset. Refer to the detailed information of the two groupings. We tested ACCstage1 using our clustering framework on M. It turned out that the groups found by running MBI 10 times for M are very consistent. With K_1_ = 100, K_2_ = 2, the consistent group corresponding to M is:

G1 = [ 2     3     4     5     6     8    10    12    15    16    17    18    19    22    26    31    35    36    37    38    43    45    50

    51    52    53    54    55    56    57    58    59    60    61    62    63    64    65    66    67    68    69    70    71    72    73

74 75 76 78 79]

G2 = [  1     7     9    11    13    14    20    21    23    24    25    27    28    29    30    32    33    34    39    40    41    42    44

    46    47    48    49    77 ]

While the consistent grouping using Y is as follows:

G1 =  [ 1     2     3     4     5     6     8     9    10    12    14    15    16    17    18    19    21    22    23    24    25    26    27

    28    31    33    34    35    36    37    38    39    42    43    45    49    50    51    52    53    54    55    56    57    58    59

    60    61    62    63    64    65    66    67    68    69    70    71    72    73    74    75    76    77    78    79]

G2 = [ 7    11    13    20    29    30    32    40    41    44    46    47    48 ]

**MBI helps identify “noise or outlier” sample from the gene expression dataset.**

1. (b)

**Figure S3.** **MBI helps identify “noise or outlier” sample from the gene expression dataset.** Comparison of the MBI clustering separation with the parameter k_2_=2 (with 2 sample groups; Figure (a)), the separation with the parameter k_2_=3 (with 3 sample groups; Figure (b)) helps remove the “noise or outlier” sample from the gene expression dataset. For this dataset of gene expression of 256 samples, there is one sample which is grouped into a separate group, Group 1. This sample is potentially an outlier sample.

**The artificial example.**

This artificial example contains three files: **bicluster_added.csv**, **bicluster.csv**, and **original.csv**.

The background original matrix (size: 20 × 20; entry values ranging from 1~100) is a rank-one matrix randomly generated in matlab; the foreground bicluster matrix (size: 20 × 20 with entry values all set to be 0, except for a bicluster of size 5×5 with entry values all set to be 10) is added to the background matrix, we get a new matrix (size: 20 × 20), which is now a rank-two matrix.

When given the bicluster_added.csv, our CSD decomposition model returns original.csv (the X matrix) and bicluster.csv (the Y matrix), which are exactly the same as given. When we test the performance of MBI on the bicluster.csv, we get **the correct bicluster of size: 5×5, with parameter setup as K_1_=20 and K_2_=2**.

The artificial example shows that our clustering framework based on CSD + MBI could effectively separate the foreground information from the background information. That is, when given the matrix of bicluster_added, it could correctly decompose it into the bicluster matrix and the original matrix.

Note that when we test the performance of NMF on the "bicluster_added.csv", NMF could not get the proper separation of the matrix.

Refer to the matrices below: **bicluster_added.csv**, **bicluster.csv**, and **original.csv**.

**bicluster_added.csv**

| 32 | 72 | 48 | 48 | 80 | 24 | 64 | 64 | 32 | 48 | 8 | 8 | 48 | 64 | 80 | 16 | 48 | 40 | 8 | 32 |
| --- | --- | --- | --- | --- | --- | --- | --- | --- | --- | --- | --- | --- | --- | --- | --- | --- | --- | --- | --- |
| 12 | 27 | 18 | 18 | 30 | 9 | 24 | 24 | 12 | 18 | 3 | 3 | 18 | 24 | 30 | 6 | 18 | 15 | 3 | 12 |
| 24 | 54 | 36 | 36 | 60 | 18 | 48 | 48 | 24 | 36 | 6 | 6 | 36 | 48 | 60 | 12 | 36 | 30 | 6 | 24 |
| 28 | 63 | 42 | 42 | 70 | 21 | 56 | 56 | 28 | 42 | 7 | 7 | 42 | 56 | 70 | 14 | 42 | 35 | 7 | 28 |
| 36 | 81 | 54 | 54 | 90 | 27 | 72 | 72 | 36 | 54 | 9 | 9 | 54 | 72 | 90 | 18 | 54 | 45 | 9 | 36 |
| 40 | 90 | 60 | 60 | 100 | 30 | 80 | 80 | 40 | 60 | 10 | 10 | 60 | 80 | 100 | 20 | 60 | 50 | 10 | 40 |
| 24 | 54 | 36 | 36 | 60 | 18 | 48 | 48 | 24 | 36 | 6 | 6 | 36 | 48 | 60 | 12 | 36 | 30 | 6 | 24 |
| 8 | 18 | 12 | 12 | 20 | 6 | 16 | 16 | 8 | 12 | 2 | 2 | 12 | 16 | 20 | 4 | 12 | 10 | 2 | 8 |
| 8 | 18 | 12 | 12 | 20 | 6 | 16 | 16 | 8 | 12 | 2 | 2 | 12 | 16 | 20 | 4 | 12 | 10 | 2 | 8 |
| 12 | 27 | 18 | 18 | 30 | 9 | 24 | 24 | 12 | ***28*** | ***13*** | ***13*** | ***28*** | ***34*** | 30 | 6 | 18 | 15 | 3 | 12 |
| 36 | 81 | 54 | 54 | 90 | 27 | 72 | 72 | 36 | ***64*** | ***19*** | ***19*** | ***64*** | ***82*** | 90 | 18 | 54 | 45 | 9 | 36 |
| 12 | 27 | 18 | 18 | 30 | 9 | 24 | 24 | 12 | ***28*** | ***13*** | ***13*** | ***28*** | ***34*** | 30 | 6 | 18 | 15 | 3 | 12 |
| 36 | 81 | 54 | 54 | 90 | 27 | 72 | 72 | 36 | ***64*** | ***19*** | ***19*** | ***64*** | ***82*** | 90 | 18 | 54 | 45 | 9 | 36 |
| 12 | 27 | 18 | 18 | 30 | 9 | 24 | 24 | 12 | ***28*** | ***13*** | ***13*** | ***28*** | ***34*** | 30 | 6 | 18 | 15 | 3 | 12 |
| 40 | 90 | 60 | 60 | 100 | 30 | 80 | 80 | 40 | 60 | 10 | 10 | 60 | 80 | 100 | 20 | 60 | 50 | 10 | 40 |
| 16 | 36 | 24 | 24 | 40 | 12 | 32 | 32 | 16 | 24 | 4 | 4 | 24 | 32 | 40 | 8 | 24 | 20 | 4 | 16 |
| 8 | 18 | 12 | 12 | 20 | 6 | 16 | 16 | 8 | 12 | 2 | 2 | 12 | 16 | 20 | 4 | 12 | 10 | 2 | 8 |
| 12 | 27 | 18 | 18 | 30 | 9 | 24 | 24 | 12 | 18 | 3 | 3 | 18 | 24 | 30 | 6 | 18 | 15 | 3 | 12 |
| 28 | 63 | 42 | 42 | 70 | 21 | 56 | 56 | 28 | 42 | 7 | 7 | 42 | 56 | 70 | 14 | 42 | 35 | 7 | 28 |
| 20 | 45 | 30 | 30 | 50 | 15 | 40 | 40 | 20 | 30 | 5 | 5 | 30 | 40 | 50 | 10 | 30 | 25 | 5 | 20 |

**original.csv**

| 32 | 72 | 48 | 48 | 80 | 24 | 64 | 64 | 32 | 48 | 8 | 8 | 48 | 64 | 80 | 16 | 48 | 40 | 8 | 32 |
| --- | --- | --- | --- | --- | --- | --- | --- | --- | --- | --- | --- | --- | --- | --- | --- | --- | --- | --- | --- |
| 12 | 27 | 18 | 18 | 30 | 9 | 24 | 24 | 12 | 18 | 3 | 3 | 18 | 24 | 30 | 6 | 18 | 15 | 3 | 12 |
| 24 | 54 | 36 | 36 | 60 | 18 | 48 | 48 | 24 | 36 | 6 | 6 | 36 | 48 | 60 | 12 | 36 | 30 | 6 | 24 |
| 28 | 63 | 42 | 42 | 70 | 21 | 56 | 56 | 28 | 42 | 7 | 7 | 42 | 56 | 70 | 14 | 42 | 35 | 7 | 28 |
| 36 | 81 | 54 | 54 | 90 | 27 | 72 | 72 | 36 | 54 | 9 | 9 | 54 | 72 | 90 | 18 | 54 | 45 | 9 | 36 |
| 40 | 90 | 60 | 60 | 100 | 30 | 80 | 80 | 40 | 60 | 10 | 10 | 60 | 80 | 100 | 20 | 60 | 50 | 10 | 40 |
| 24 | 54 | 36 | 36 | 60 | 18 | 48 | 48 | 24 | 36 | 6 | 6 | 36 | 48 | 60 | 12 | 36 | 30 | 6 | 24 |
| 8 | 18 | 12 | 12 | 20 | 6 | 16 | 16 | 8 | 12 | 2 | 2 | 12 | 16 | 20 | 4 | 12 | 10 | 2 | 8 |
| 8 | 18 | 12 | 12 | 20 | 6 | 16 | 16 | 8 | 12 | 2 | 2 | 12 | 16 | 20 | 4 | 12 | 10 | 2 | 8 |
| 12 | 27 | 18 | 18 | 30 | 9 | 24 | 24 | 12 | ***18*** | ***3*** | ***3*** | ***18*** | ***24*** | 30 | 6 | 18 | 15 | 3 | 12 |
| 36 | 81 | 54 | 54 | 90 | 27 | 72 | 72 | 36 | ***54*** | ***9*** | ***9*** | ***54*** | ***72*** | 90 | 18 | 54 | 45 | 9 | 36 |
| 12 | 27 | 18 | 18 | 30 | 9 | 24 | 24 | 12 | ***18*** | ***3*** | ***3*** | ***18*** | ***24*** | 30 | 6 | 18 | 15 | 3 | 12 |
| 36 | 81 | 54 | 54 | 90 | 27 | 72 | 72 | 36 | ***54*** | ***9*** | ***9*** | ***54*** | ***72*** | 90 | 18 | 54 | 45 | 9 | 36 |
| 12 | 27 | 18 | 18 | 30 | 9 | 24 | 24 | 12 | ***18*** | ***3*** | ***3*** | ***18*** | ***24*** | 30 | 6 | 18 | 15 | 3 | 12 |
| 40 | 90 | 60 | 60 | 100 | 30 | 80 | 80 | 40 | 60 | 10 | 10 | 60 | 80 | 100 | 20 | 60 | 50 | 10 | 40 |
| 16 | 36 | 24 | 24 | 40 | 12 | 32 | 32 | 16 | 24 | 4 | 4 | 24 | 32 | 40 | 8 | 24 | 20 | 4 | 16 |
| 8 | 18 | 12 | 12 | 20 | 6 | 16 | 16 | 8 | 12 | 2 | 2 | 12 | 16 | 20 | 4 | 12 | 10 | 2 | 8 |
| 12 | 27 | 18 | 18 | 30 | 9 | 24 | 24 | 12 | 18 | 3 | 3 | 18 | 24 | 30 | 6 | 18 | 15 | 3 | 12 |
| 28 | 63 | 42 | 42 | 70 | 21 | 56 | 56 | 28 | 42 | 7 | 7 | 42 | 56 | 70 | 14 | 42 | 35 | 7 | 28 |
| 20 | 45 | 30 | 30 | 50 | 15 | 40 | 40 | 20 | 30 | 5 | 5 | 30 | 40 | 50 | 10 | 30 | 25 | 5 | 20 |

**bicluster.csv**

| 0 | 0 | 0 | 0 | 0 | 0 | 0 | 0 | 0 | 0 | 0 | 0 | 0 | 0 | 0 | 0 | 0 | 0 | 0 | 0 |
| --- | --- | --- | --- | --- | --- | --- | --- | --- | --- | --- | --- | --- | --- | --- | --- | --- | --- | --- | --- |
| 0 | 0 | 0 | 0 | 0 | 0 | 0 | 0 | 0 | 0 | 0 | 0 | 0 | 0 | 0 | 0 | 0 | 0 | 0 | 0 |
| 0 | 0 | 0 | 0 | 0 | 0 | 0 | 0 | 0 | 0 | 0 | 0 | 0 | 0 | 0 | 0 | 0 | 0 | 0 | 0 |
| 0 | 0 | 0 | 0 | 0 | 0 | 0 | 0 | 0 | 0 | 0 | 0 | 0 | 0 | 0 | 0 | 0 | 0 | 0 | 0 |
| 0 | 0 | 0 | 0 | 0 | 0 | 0 | 0 | 0 | 0 | 0 | 0 | 0 | 0 | 0 | 0 | 0 | 0 | 0 | 0 |
| 0 | 0 | 0 | 0 | 0 | 0 | 0 | 0 | 0 | 0 | 0 | 0 | 0 | 0 | 0 | 0 | 0 | 0 | 0 | 0 |
| 0 | 0 | 0 | 0 | 0 | 0 | 0 | 0 | 0 | 0 | 0 | 0 | 0 | 0 | 0 | 0 | 0 | 0 | 0 | 0 |
| 0 | 0 | 0 | 0 | 0 | 0 | 0 | 0 | 0 | 0 | 0 | 0 | 0 | 0 | 0 | 0 | 0 | 0 | 0 | 0 |
| 0 | 0 | 0 | 0 | 0 | 0 | 0 | 0 | 0 | 0 | 0 | 0 | 0 | 0 | 0 | 0 | 0 | 0 | 0 | 0 |
| 0 | 0 | 0 | 0 | 0 | 0 | 0 | 0 | 0 | **10** | **10** | **10** | **10** | **10** | 0 | 0 | 0 | 0 | 0 | 0 |
| 0 | 0 | 0 | 0 | 0 | 0 | 0 | 0 | 0 | **10** | **10** | **10** | **10** | **10** | 0 | 0 | 0 | 0 | 0 | 0 |
| 0 | 0 | 0 | 0 | 0 | 0 | 0 | 0 | 0 | **10** | **10** | **10** | **10** | **10** | 0 | 0 | 0 | 0 | 0 | 0 |
| 0 | 0 | 0 | 0 | 0 | 0 | 0 | 0 | 0 | **10** | **10** | **10** | **10** | **10** | 0 | 0 | 0 | 0 | 0 | 0 |
| 0 | 0 | 0 | 0 | 0 | 0 | 0 | 0 | 0 | **10** | **10** | **10** | **10** | **10** | 0 | 0 | 0 | 0 | 0 | 0 |
| 0 | 0 | 0 | 0 | 0 | 0 | 0 | 0 | 0 | 0 | 0 | 0 | 0 | 0 | 0 | 0 | 0 | 0 | 0 | 0 |
| 0 | 0 | 0 | 0 | 0 | 0 | 0 | 0 | 0 | 0 | 0 | 0 | 0 | 0 | 0 | 0 | 0 | 0 | 0 | 0 |
| 0 | 0 | 0 | 0 | 0 | 0 | 0 | 0 | 0 | 0 | 0 | 0 | 0 | 0 | 0 | 0 | 0 | 0 | 0 | 0 |
| 0 | 0 | 0 | 0 | 0 | 0 | 0 | 0 | 0 | 0 | 0 | 0 | 0 | 0 | 0 | 0 | 0 | 0 | 0 | 0 |
| 0 | 0 | 0 | 0 | 0 | 0 | 0 | 0 | 0 | 0 | 0 | 0 | 0 | 0 | 0 | 0 | 0 | 0 | 0 | 0 |
| 0 | 0 | 0 | 0 | 0 | 0 | 0 | 0 | 0 | 0 | 0 | 0 | 0 | 0 | 0 | 0 | 0 | 0 | 0 | 0 |

**References**

1. Lee M, Shen H, Huang JZ, Marron JS (2010) Biclustering via sparse singular value decomposition. Biometrics 66: 1087-95.
2. Liu Y, Hayes DN, Nobel A & Marron JS (2008) [Statistical significance of clustering for high dimension low sample size data](http://www.unc.edu/~yfliu/papers/sigclust.pdf). J Am Stat Accoc. 103: 1281-1293.
3. Bhattacharjee A, Richards WG, Staunton J, Li C, Monti S, et al. (2001) Classification of human lung carcinomas by mRNA expression profiling reveals distinct adenocarcinoma subclasses. Proc Natl Acad Sci USA 98: 13790-13795.
4. Wilkerson MD, Yin X, Walter V, Zhao N, Cabanski CR, et al. (2012) Differential pathogenesis of lung adenocarcinoma subtypes involving sequence mutations, copy number, chromosomal instability and methylation. PLoS One 7.
5. Shedden K, Taylor JM, Enkemann SA, Tsao MS, Yeatman TJ, et al. (2008) Gene expression-based survival prediction in lung adenocarcinoma: a multi-site, blinded validation study. Nat Med 14: 822–827.
6. Tomida S, Takeuchi T, Shimada Y, Arima C, Matsuo K, et al. (2009) Relapse-related molecular signature in lung adenocarcinomas identifies patients with dismal prognosis. J Clin Oncol 27: 2793-9.
7. Bryant CM, Albertus DL, Kim S, Chen G, Brambilla C, et al. (2010) Clinically Relevant Characterization of Lung Adenocarcinoma Subtypes Based on Cellular Pathways: An International Validation Study. PLoS ONE 5(7): e11712.
8. Wilkerson, M.D. & Hayes, D.N. (2010) ConsensusClusterPlus: a class discovery tool with confidence assessments and item tracking. Bioinformatics 26: 1572–1573.
9. Tibshirani, R., Hastie, T., Narasimhan, B. & Chu, G. (2002) Diagnosis of multiple cancer types by shrunken centroids of gene expression. Proc Natl Acad Sci 99: 6567–6572. [

](http://www.biomedcentral.com/sfx_links?ui=1471-2199-9-79&bibl=B4)
